# Effects of daridorexant on sleep architecture in Japanese patients with insomnia disorder: analysis of a phase II randomized controlled trial

**DOI:** 10.1007/s41105-025-00628-2

**Published:** 2026-02-09

**Authors:** Tomoko Yagi, Motohiro Ozone, Tetsuya Ioji, Kenta Murotani, Akinori Nishi, Naohisa Uchimura

**Affiliations:** 1 School of Medical Technology, Kurume University School of Medicine, 777-1 Higashikushihara-machi, Fukuoka 830-0003 Kurume, Japan; 2 Department of Neuropsychiatry, Kurume University School of Medicine, 67 Asahi-machi, Fukuoka 830-0011 Kurume, Japan; 3 Bio-Statistics Center, Kurume University, 67 Asahi-machi, Fukuoka 830-0011 Kurume, Japan

**Keywords:** Daridorexant, Insomnia disorder, Sleep architecture, Polysomnography, Persistent awakening

## Abstract

While daridorexant, a dual orexin receptor antagonist (DORA), demonstrated dose-dependent improvements in sleep parameters in a Japanese Phase II trial, its effects on sleep architecture remain incompletely characterized. This secondary analysis examined daridorexant’s effects on sleep fragmentation and architecture in Japanese patients with insomnia disorder. Forty-seven Japanese patients with insomnia (mean age 50.4 ± 8.0 years) underwent 10 nights of polysomnographic (PSG) recordings in a randomized protocol (baseline, placebo, daridorexant 10 mg, 25 mg, 50 mg; 2 nights each). Key parameters included latency to persistent sleep (LPS), total sleep time (TST), wake after sleep onset (WASO), sleep stage durations, and the number of persistent awakenings (NAW). The 8-hour recording was divided into quarters for temporal analysis. Dose-response trends were assessed using Jonckheere-Terpstra tests. Significant dose-response relationships were observed for LPS (*P* = 0.004), TST (*P* < 0.001), WASO (*P* < 0.001), and NAW (*P* = 0.004). Changes from baseline showed dose-dependent reductions in N1 time (*P* = 0.006) and increases in N2 (*P* = 0.049), N3 (*P* = 0.050), and REM sleep times (*P* < 0.001). Quartile analysis revealed a significant reduction in NAW in the first quarter with 50 mg (*P* = 0.012) and a significant increase in REM sleep in the first and fourth quarters with 25 mg and 50 mg (*P* < 0.05). Daridorexant dose-dependently improves sleep architecture by reducing fragmentation and enhancing deeper NREM and REM sleep. Temporal analysis demonstrated sustained effects throughout the night with REM sleep enhancement persisting through the final quarter. These findings suggest restoration of natural sleep architecture supporting daridorexant’s potential as a comprehensive sleep-promoting agent.

**Supplementary Information:**

The online version contains supplementary material available at 10.1007/s41105-025-00628-2.

## Introduction

Insomnia impairs quality of life in daily activities and has been identified as a risk factor for the development of depression [1–4]. Additionally, patients with insomnia have a higher prevalence of diabetes and hypertension, and insomnia is increasingly recognized as a contributing factor to these conditions [5].

A 2022 meta-analysis [6] reported that approximately 53% of individuals experienced subjective insomnia symptoms, while approximately 17% were clinically diagnosed with insomnia disorder. Although cognitive behavioral therapy is becoming more widespread, pharmacotherapy remains the mainstay of insomnia treatment. Hypnotics have evolved from barbiturates to benzodiazepines (BZ) and non-benzodiazepines (non-BZ). However, concerns regarding long-term use, including dependence and abuse, have led to increased interest in agents with alternative mechanisms of action, such as melatonin receptor agonists and dual orexin receptor antagonists (DORAs).

Daridorexant is the third DORA approved in the United States and Japan, following suvorexant and lemborexant, and the first in Europe. This agent competitively and reversibly inhibits the binding of orexin [7, 8], a neurotransmitter that maintains wakefulness, to its receptors (orexin-1 and orexin-2), thereby suppressing excessive arousal and facilitating the physiological transition from wakefulness to sleep. In the development of daridorexant, a Phase II clinical trial was conducted in Japan (Japan Registry of Clinical Trials: jRCT2080224596), which demonstrated statistically significant dose-response relationships for objective measures using polysomnography, including latency to persistent sleep and wake after sleep onset [9].

In this study, we performed statistical analysis using polysomnographic (PSG) data collected from the Phase II clinical trial to clarify the effects of daridorexant on sleep architecture, including sleep fragmentation and non-REM and REM sleep parameters. Specifically, we evaluated the efficacy of daridorexant administration by examining whether dose-response relationships could be identified using change from baseline as the effect size.

## Methods

### Subjects

The study population comprised PSG data from 47 patients with insomnia disorder who met the inclusion criteria of the Phase II trial. Inclusion criteria were: Japanese individuals aged 16 to 64 years with subjective insomnia symptoms (sleep onset latency ≥ 30 min, nocturnal awakenings ≥ 30 min, or total sleep time ≤ 6.5 h) occurring at least 3 nights per week for at least 3 months and meeting DSM-5 criteria for insomnia disorder. Participants demonstrated objective sleep disturbances on two nights of baseline PSG recordings: mean sleep onset latency ≥ 20 min (neither night < 15 min), mean wake after sleep onset ≥ 30 min (neither night < 20 min), and mean total sleep time < 420 min. Participants had no other sleep disorders including sleep apnea syndrome, periodic limb movement disorder, restless legs syndrome, circadian rhythm disorders, or REM sleep behavior disorder. Baseline characteristics were: 22 males and 25 females, mean age 50.4 ± 8.0 years, BMI 22.7 ± 2.5 kg/m², duration of insomnia disorder 8.2 ± 8.2 years, and Insomnia Severity Scale score 19.4 ± 3.1 points.

### Data acquisition

**Fig. 1:**
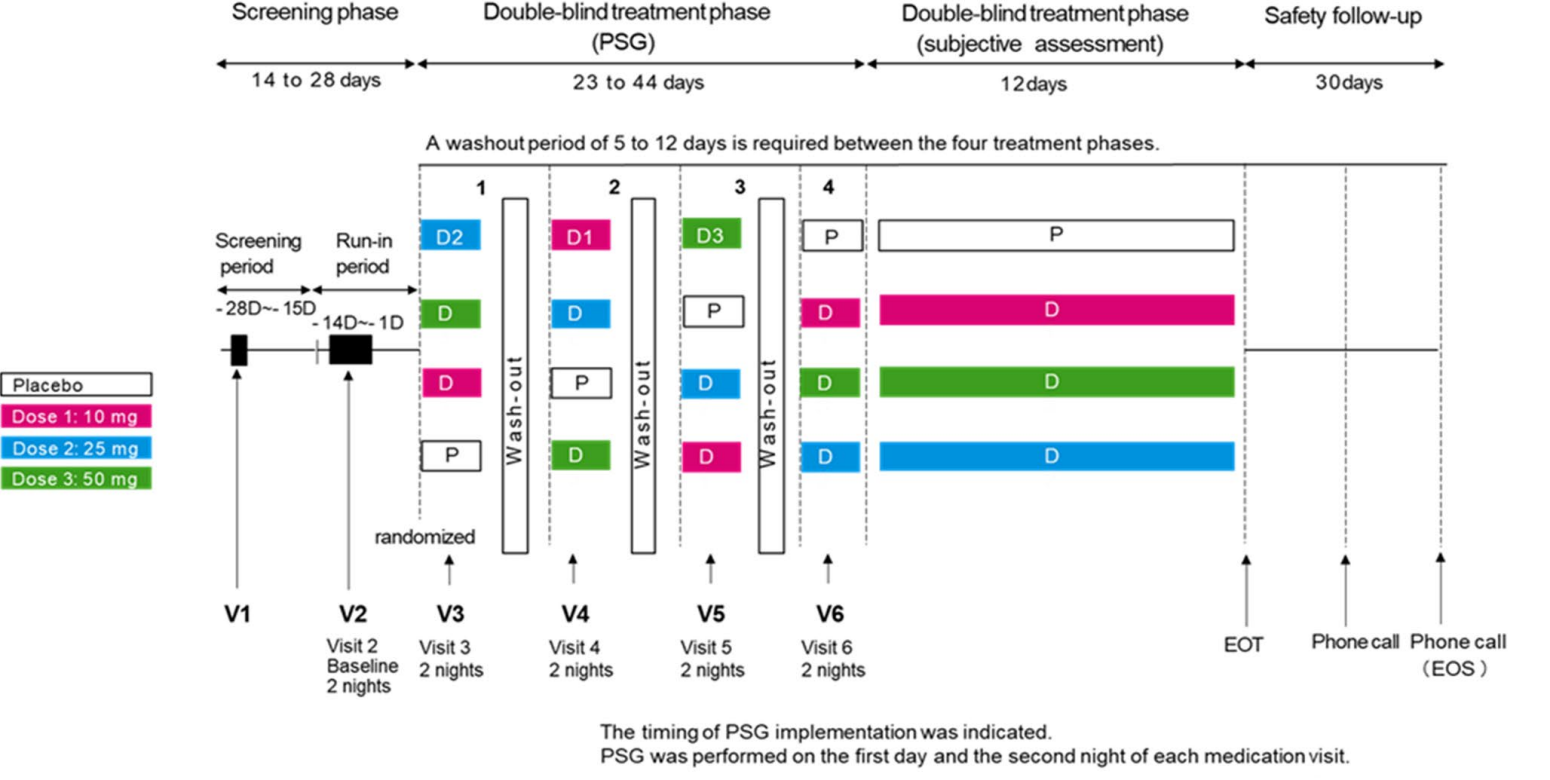
Study design and schedule of PSG assessments: baseline (visit 2) and randomized treatment periods (visits 3–6). Study design and polysomnographic assessment schedule for the Phase II clinical trial of daridorexant. The trial employed a randomized, double-blind, placebo-controlled, 4-period crossover design. Following baseline assessment (Visit 2, 2 nights), each participant underwent four treatment periods (Visits 3–6, 2 nights each) receiving placebo, daridorexant 10 mg, 25 mg, and 50 mg in randomized sequence. Polysomnographic recordings were conducted for 8 h per night under standardized laboratory conditions. Each treatment period was separated by appropriate washout intervals to prevent carryover effects. The crossover design ensured that each participant served as their own control, minimizing inter-individual variability and maximizing statistical power for dose-response analysis. All PSG data underwent central blinded scoring by qualified sleep technologists according to AASM guidelines. PSG = polysomnography; AASM = American Academy of Sleep Medicine

In the Phase II clinical trial, each participant underwent PSG recordings for a total of 10 nights: 2 nights at baseline (visit 2) and 2 nights during each of 4 randomly assigned treatment periods (visits 3–6, allocated to placebo, daridorexant 10 mg, 25 mg, and 50 mg) (Fig. 1). A total of 470 PSG datasets were obtained from 47 participants.

PSG recordings simultaneously measured electroencephalography (EEG), electrooculography, electromyography of the chin muscles, and electrocardiography for 8 h per night. PSG data recorded at each site were blinded and subsequently underwent visual scoring by registered polysomnographic technologists specifically enrolled for this trial.

### Data scoring and sleep parameters

Sleep stage scoring was performed according to the American Academy of Sleep Medicine Scoring Manual version 2.3. Scoring was conducted by 6 technologists, including the central scoring supervisor and 5 experienced polysomnographic technologists. Prior to study initiation, inter-rater reliability validation was performed to ensure scoring accuracy, requiring intra-rater reproducibility accuracy of ≥ 90% and inter-rater agreement rates of ≥ 85%. Following scoring, quantitative parameters were calculated as follows: total sleep time (TST, min), defined as the sum of non-wake stages during the 8-hour recording period; latency to persistent sleep (LPS, min), defined as the time from lights-out to the first epoch of 20 consecutive non-wake stage epochs; and wake after sleep onset (WASO, min), defined as the total duration of stage W from persistent sleep onset to the final sleep epoch. To evaluate sleep fragmentation, the number of persistent awakenings (NAW, n) was defined as awakenings lasting 2 or more consecutive stage W epochs and terminated by stages N2, N3, or R. Qualitative parameters included the duration and percentage of non-REM sleep stages (N1, N2, N3) and REM sleep. Furthermore, to analyze temporal changes in sleep parameters throughout the night, each parameter was calculated for four quarters of the total recording time (TRT) of 8 h: first quarter (0–2 h post lights-out), second quarter (2–4 h), third quarter (4–6 h), and fourth quarter (6–8 h to lights-on).

### Statistical analysis methods

For the four treatment periods with four dose groups (placebo, daridorexant 10 mg, 25 mg, 50 mg) with 2 nights per visit, parameters were calculated using the mean values of the 2 nights. Dose-response relationships were analyzed using the Jonckheere-Terpstra trend test for mean values between baseline and the four dose groups. Similarly, dose-response relationships were analyzed using the Jonckheere-Terpstra trend test for the change from baseline for each of the four dose groups. Furthermore, to examine how long the drug effects persisted throughout the night compared to placebo, differences between the change from baseline for the three active dose groups (10 mg, 25 mg, 50 mg) and the change from baseline for placebo were tested using adjusted p-values by Dunnett’s method for parameters in each of the four quarters of the night. All analyses were performed with a two-sided significance level of 5%. Statistical analysis was conducted using SAS 9.4 (SAS Institute, Cary, NC, USA).

### Clinical trials registration

This study is a secondary analysis of data from a Phase II clinical trial of daridorexant [(Japan Registry of Clinical Trials, jRCT2080224596 (https://jrct.niph.go.jp/latest-detail/jRCT2080224596)].

### Ethical considerations

In this study, anonymized PSG parameter data were provided by Idorsia Pharmaceuticals Japan Ltd. (currently Nexera Pharma Japan Ltd.), and no subject identification information was included. This research was reviewed by the Ethics Committee for Medical Research at Kurume University for appropriateness of study conduct and received approval on June 26, 2024 (Research Number: 24081).

## Results

### Polysomnographic parameters at baseline and during treatment with placebo and daridorexant

Table 1 presents the PSG parameters for the 47 patients with insomnia disorder at baseline and during treatment with placebo, daridorexant (10 mg, 25 mg, 50 mg). At baseline, time-related parameters showed latency to persistent sleep (LPS) of 57.0 ± 47.7 min, wake after sleep onset (WASO) of 84.2 ± 40.5 min, and total sleep time (TST) of 349.6 ± 53.9 min, indicating prolonged LPS, increased WASO, and shortened TST. With 50 mg treatment, LPS decreased to 13.1 ± 9.5 min, WASO decreased to 33.3 ± 29.7 min, and TST increased to 437.3 ± 32.1 min, with significant dose-response relationships observed (LPS: *P* = 0.004, WASO: *P* < 0.001, TST: *P* < 0.001). The number of persistent awakenings (NAW) decreased from 10.0 ± 4.3 episodes at baseline to 6.2 ± 3.5 episodes during 50 mg treatment, with significant dose-dependent decreases (*P* = 0.004).

Regarding sleep stages, baseline values showed stage N1 66.9 ± 34.9 min, stage N2 172.4 ± 38.5 min, stage N3 39.2 ± 28.6 min, and stage R 71.1 ± 24.4 min. Stage-specific percentages were: N1 20.1 ± 11.0%, N2 49.1 ± 9.1%, N3 11.2 ± 8.1%, and R 19.6 ± 6.3%, indicating excessive N1 sleep and reduced N2 and N3 sleep, and REM sleep. During 50 mg treatment, stage N1 decreased to 58.4 ± 27.6 min, while stage N2 increased to 209.1 ± 32.7 min, stage N3 increased to 50.5 ± 32.9 min, and stage R increased to 119.2 ± 26.0 min. Significant dose-response relationships were observed for increased stage R time (*P* < 0.001), decreased %stage N1 (*P* = 0.016), and increased %stage R (*P* < 0.001).

**Table 1:** Polysomnographic parameters at baseline and during treatment with placebo and Daridorexant in Japanese patients with insomnia disorder

PSG parameter	Baseline (*n*=47)	Placebo (*n*=47)	10 mg (*n*=47)	25 mg (*n*=47)	50 mg (*n*=47)	*p*-value†
*Sleep continuity parameters*						
Total sleep time, min	349.6±53.9	402.4±39.8	420.3±26.5	426.3±26.9	437.3±32.1	<0.001*
Wake after sleep onset, min	84.2±40.5	61.2±34.1	46.2±23.7	41.2±24.4	33.3±29.7	<0.001*
Latency to persistent sleep, min	57.0±47.7	21.4±17.3	17.5±12.3	16.2±11.3	13.1±9.5	0.004*
Number of persistent awakening, n	10.0±4.3	7.9±3.6	7.7±3.9	7.6±4.1	6.2±3.5	0.004*
*Sleep Stage Durations*,* min*						
Stage N1 time	66.9±34.9	67.5±32.6	64.1±31.7	63.0±30.1	58.4±27.6	0.137
Stage N2 time	172.4±38.5	201.5±35.1	207.9±35.9	209.6±34.2	209.1±32.7	0.069
Stage N3 time	39.2±28.6	44.5±32.9	47.2±32.0	46.7±33.6	50.5±32.9	0.196
Total NREM time	278.5±44.3	313.5±34.0	319.1±31.0	319.3±32.4	318.0±29.4	0.189
Stage R time	71.1±24.4	88.9±26.6	101.1±25.8	106.9±25.7	119.2±26.0	<0.001*
*Sleep Stage Percentages*,* %*						
Stage N1	20.1±11.0	17.1±7.9	15.3±7.5	14.9±7.2	13.7±7.3	0.016*
Stage N2	49.1±9.1	50.0±6.8	49.3±7.4	49.0±6.8	47.7±6.5	0.092
Stage N3	11.2±8.1	11.1±8.0	11.3±7.7	11.0±7.8	11.5±7.3	0.387
Total NREM	80.4±6.3	78.2±5.9	76.0±6.0	75.0±5.8	72.9±5.5	<0.001*
Stage R	19.6±6.3	21.8±5.9	24.0±6.0	25.0±5.8	27.1±5.5	<0.001*

Data are presented as mean ± standard deviation. †P-values from Jonckheere-Terpstra trend test for dose-response relationships. *Statistically significant at *P* < 0.05. *PSG* polysomnography; *NREM* non-rapid eye movement; *R* rapid eye movement

### Changes from baseline in polysomnographic parameters with placebo and daridorexant treatment: dose-response analysis

**Fig. 2:**
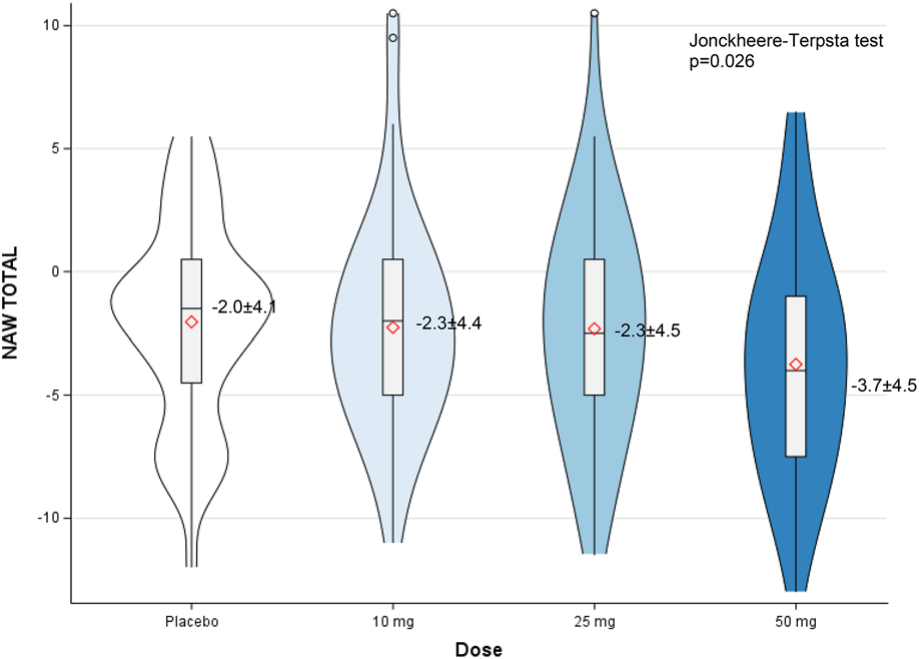
Dose-response relationship for changes in number of persistent awakenings. Dose-response relationship for changes in number of persistent awakenings (NAW) from baseline. Data are presented as mean ± standard error of the mean (SEM). Statistical analysis performed using Jonckheere-Terpstra trend test. *P = 0.026 indicates significant dose-response relationship across treatment groups. Negative values indicate reduction from baseline.* NAW* number of persistent awakenings;* DR* daridorexant

Table 2 shows the results of dose-response relationship analysis for changes from baseline. Significant dose-response relationships were observed for TST (placebo = 52.8 ± 48.7 min, 10 mg = 70.7 ± 52.3 min, 25 mg = 76.6 ± 44.2 min, 50 mg = 87.6 ± 42.8 min, *P* < 0.001) and WASO (placebo = -23.0 ± 38.0 min, 10 mg = -38.0 ± 43.1 min, 25 mg = -43.0 ± 34.6 min, 50 mg = -50.9 ± 30.7 min, *P* < 0.001). NAW also showed significant dose-response relationships (placebo = -2.0 ± 4.1 n, 10 mg = -2.3 ± 4.4 n, 25 mg = -2.3 ± 4.5 n, 50 mg = -3.7 ± 4.5 n, *P* = 0.026) (Fig. 2).

**Fig. 3:**
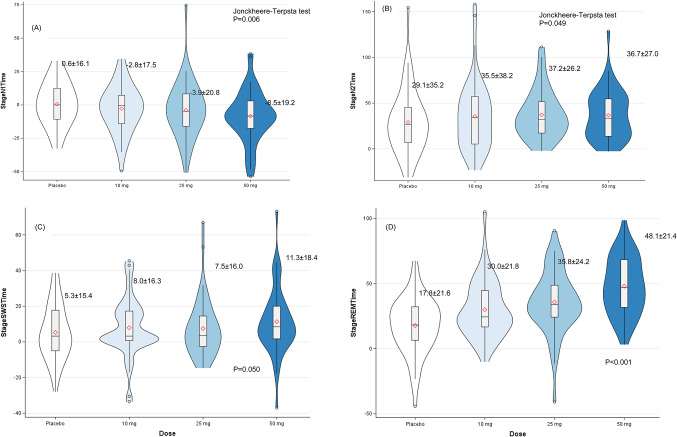
Changes from baseline in sleep stage durations by treatment group. Changes from baseline in sleep stage durations by treatment group.** A** Stage N1 sleep time,** B** Stage N2 sleep time,** C** Stage N3 sleep time,** D** REM sleep time. Data are presented as mean ± standard error of the mean from 47 participants per group. Jonckheere-Terpstra trend tests demonstrated significant dose-response relationships for all parameters: Stage N1 (*P* = 0.006, progressive decrease indicating reduced light sleep), Stage N2 (*P* = 0.049, progressive increase), Stage N3 (*P* = 0.050, progressive increase indicating enhanced deep sleep), and REM sleep (*P* < 0.001, robust progressive increase). Results demonstrate daridorexant’s beneficial restructuring of sleep architecture with reduction of superficial sleep and enhancement of restorative sleep stages. Positive values indicate increases from baseline; negative values indicate decreases from baseline.* DR* daridorexant,* REM*  rapid eye movement sleep

For changes in sleep stage durations, stage N1 time showed significant dose-dependent decreases (placebo = 0.6 ± 16.1 min, 10 mg = -2.8 ± 17.5 min, 25 mg = -3.9 ± 20.8 min, 50 mg = -8.5 ± 19.2 min, *P* = 0.006), while stage N2, N3 and R times showed significant dose-dependent increases (N2: placebo = 29.1 ± 35.2 min, 50 mg = 36.7 ± 27.0 min, *P* = 0.049; N3: placebo = 5.2 ± 15.4 min, 50 mg = 11.3 ± 18.4 min, *P* = 0.050; R: placebo = 17.8 ± 21.6 min, 50 mg = 48.1 ± 21.4 min, *P* < 0.001) (Fig. 3).

**Table 2:** Changes from baseline in polysomnographic parameters with placebo and Daridorexant treatment: Dose-response analysis

PSG parameter	placebo (*n*=47)	10 mg (*n*=47)	25 mg (*n*=47)	50 mg (*n*=47)	*p*-value†
*Sleep continuity parameters*					
Total sleep time, min	52.8±48.7	70.7±52.3	76.6±44.2	87.6±42.8	<0.001*
Wake after sleep onset, min	-23.0±38.0	-38.0±43.1	-43.0±34.6	-50.9±30.7	<0.001*
Latency to persistent sleep, min	-35.6±43.1	-39.5±46.0	-40.8±44.5	-43.9±45.8	0.092
Number of persistent awakening, n	-2.0±4.1	-2.3±4.4	-2.3±4.5	-3.7±4.5	0.026*
*Sleep Stage Durations*,* min*					
Stage N1 time	0.6±16.1	-2.8±17.5	-3.9±20.8	-8.5±19.2	0.006*
Stage N2 time	29.1±35.2	35.5±38.2	37.2±26.2	36.7±27.0	0.049*
Stage N3 time	5.3±15.4	8.0±16.3	7.5±16.0	11.3±18.4	0.050*
Total NREM time	35.0±39.0	40.7±42.8	40.8±37.7	39.6±36.2	0.16
Stage R time	17.8±21.6	30.0±21.8	35.8±24.2	48.1±21.4	<0.001*
*Sleep Stage Percentages per TST*,* %*					
Stage N1	-3.0±6.2	-4.8±7.0	-5.2±6.4	-6.4±5.6	<0.001*
Stage N2	0.9±6.2	0.3±6.3	-0.1±5.5	-1.4±6.0	0.016*
Stage N3	0.0±4.0	0.2±4.3	-0.1±3.8	0.4±4.6	0.42
Total NREM	-2.2±4.8	-4.3±4.7	-5.4±5.4	-7.5±4.6	<0.001*
Stage R time	2.2±4.8	4.3±4.7	5.4±5.4	7.5±4.6	<0.001*

Data are presented as mean ± standard deviation of change from baseline. †P-values from Jonckheere-Terpstra trend test for dose-response relationships across placebo, 10 mg, 25 mg, and 50 mg daridorexant. *Statistically significant at *P* < 0.05. Positive values indicate increases from baseline; negative values indicate decreases from baseline. *PSG* polysomnography, *NREM* non-rapid eye movement, *R* rapid eye movement

### Temporal analysis in sleep parameters throughout the night

**Fig. 4:**
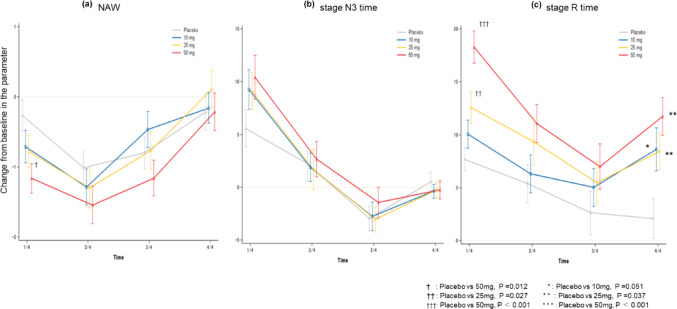
Quartile-based changes in the parameter (number of persistent awakenings, stage N3 time, stage R time) from baseline across treatment groups. Changes from baseline in sleep parameters during four quarters of the night.** A** Number of persistent awakenings (NAW),** B** Stage N3 sleep time,** C** REM sleep time. Each quarter represents 2 h of the 8-hour recording period (1/4: 0–2 h, 2/4: 2–4 h, 3/4: 4–6 h, 4/4: 6–8 h). Data are presented as mean ± standard error of the mean (SEM). Statistical comparisons between active doses and placebo using Dunnett’s method (detailed P-values in Appendix A, Tables 3, 4 and 5). Significant differences from placebo: ***P < 0.001, **P < 0.01, *P < 0.05.* DR* daridorexant, *NAW *  number of persistent awakenings;* REM*  rapid eye movement sleep,* SEM*  standard error of the mean

Changes from baseline for NAW, stage N3 time, and stage R time were examined across the four quarters of the night (Fig. 4). For NAW (Fig. 4a), the active treatment groups showed greater reductions compared to placebo during the first and second quarters, with 50 mg showing continued reduction through the third quarter. Statistically significant differences from placebo were observed for 50 mg during the first quarter (placebo = -0.3 ± 0.2 n vs. 50 mg = -1.2 ± 0.2 n, *P* = 0.012).

For stage N3 time (Fig. 4b), the active treatment groups showed greater increases compared to placebo during the first quarter. For stage R time (Fig. 4c), the active treatment groups showed greater increases during the first quarter, with 50 mg showing greater increases. Notably, while placebo showed decreased changes in the final quarter, the active treatment groups showed increased changes. Statistically significant differences from placebo were observed for stage R time during the first quarter (placebo = 7.7 ± 1.1 min vs. 25 mg = 12.6 ± 1.5 min, *P* = 0.027, 50 mg = 18.3 ± 1.5 min, *P* < 0.001) and fourth quarter (placebo = 2.1 ± 1.9 min vs. 25 mg = 8.4 ± 1.7 min, *P* = 0.037, 50 mg = 11.7 ± 1.8 min, *P* < 0.001).

## Discussion

### Overview of hypnotic effects on sleep architecture

BZ and non-BZ hypnotics, which have been used as sleep medications for over half a century, exert hypnotic effects by acting on GABA receptors. Their known effects on PSG include shortened sleep onset latency, reduced slow-wave sleep, increased sleep spindles, and decreased REM sleep [10–12]. In Japan and the United States, orexin receptor antagonists have been available for 10 years, and due to their high safety profile and weak dependence potential [13–16], they are increasingly used as first-line sleep medications, with growing recognition among general practitioners. However, there are few systematic reports on how these agents affect human sleep architecture.

The PSG data analysis results from daridorexant administration to insomnia patients in this study demonstrated dose-dependent improvement in multiple sleep parameters.

### Previous PSG studies of orexin receptor antagonists

Clinical trials using PSG with various orexin receptor antagonists [9, 17, 18] have reported sleep effects including shortened latency to persistent sleep and prolonged total sleep time. Furthermore, a systematic review reported that prolonged total sleep time and increased REM sleep time were common findings for DORAs in both healthy subjects and insomnia patients. In this Phase II clinical trial, both parameter values during each treatment period and changes from baseline showed prolonged total sleep time and increased REM sleep time and percentage. However, the effects of DORA on NREM sleep remain inconclusive, with most insomnia studies reporting no significant effects on total NREM sleep, though some show decreased %stage N1 and variable effects on %stage N2 and N3.

### Effects of daridorexant on NREM sleep architecture

One notable finding in this study is the effect of daridorexant administration on NREM sleep architecture. Although no significant dose-dependent relationship was observed for total NREM sleep duration, an increase of approximately 40 min was observed from baseline (278.5 ± 44.3 min) to DR 25 mg treatment (319.3 ± 32.4 min). Regarding stage-specific NREM sleep durations, stage N1 showed statistically significant dose-dependent decreases in change from baseline, while stage N2 and stage N3 showed statistically significant increases. The significance of these findings was confirmed using the Jonckheere-Terpstra test, indicating clear changes in sleep architecture between placebo and active treatment. Therefore, daridorexant administration in insomnia reduced light NREM sleep while increasing deeper NREM sleep (stages N2 and N3), suggesting that improvement in NREM sleep architecture can be expected in insomnia patients.

### Effects of daridorexant-induced reduction in nocturnal awakenings on sleep architecture

In this study, a dose-dependent relationship was observed between the reduction in the number of persistent nocturnal awakenings and daridorexant dose. A previous post-hoc analysis of daridorexant Phase 2 data showed that long awakenings (≥ 6 min) were significantly reduced with 50 mg compared to placebo [19]. In the sleep quarter analysis, NAW (Fig. 4a) showed greater reductions compared to placebo during the first two quarters of sleep for all three doses, with 50 mg showing continued reduction through the third quarter. Statistically, 50 mg demonstrated a significant effect in reducing awakenings during the first quarter of sleep.

The persistent awakenings (NAW) defined in this study indicate disruption of sleep stage continuity, differing from brief arousal responses. NAW was defined as awakenings lasting at least 1 min (2 consecutive epochs of stage W). These awakenings require subsequent sleep onset beginning with light sleep, suggesting that the number of sustained awakenings indicates fragmentation and superficiality of sleep structure. Therefore, the dose-dependent relationship observed for NAW in this analysis is presumed to be related to daridorexant-induced structural changes in NREM sleep (reduction of light sleep and increase of deeper sleep). Furthermore, awakenings with persistent durations of 5 min or longer are considered memorable [20], and their reduction would be effective for improving insomnia symptoms.

### Slow wave sleep (SWS) enhancement effects

In this study, it was presumed that the reduction of persistent awakenings led to deeper sleep, with significant dose-dependent relationships observed not only for N2 increases but also for N3 increases. In the sleep quarter analysis, N3 time (Fig. 4b) showed greater increases compared to placebo during the first quarter of sleep for all three doses. Enhancement of deep sleep by DORA has been reported in basic research using rats and mice [21, 22]. In both young adult rats with sleep fragmentation caused by ethanol vapor exposure and control rats, DORA-12 administration increased delta and theta power in sleep EEG, confirming increased deeper sleep [21]. This study represents the first report showing daridorexant tends to increase SWS sleep during the first quarter of sleep in humans.

### REM sleep enhancement effects

According to a review of DORA effects on REM sleep, REM sleep latency shortening was observed in 60–70% of studies, and REM sleep amount increases were observed in over 80% of studies targeting insomnia patients. In this study, significant dose-dependent relationships were observed for REM sleep latency, REM sleep time, and REM sleep percentage in patients with objective insomnia on PSG. Since the baseline REM sleep percentage before medication was 19.6 ± 6.3%, below 20%, the involvement of REM sleep rebound phenomenon [23] in insomnia was presumed to contribute to this REM sleep increase. The sleep quarter analysis (Fig. 4c) showed significant increases compared to placebo during the first quarter of sleep with 25 mg and 50 mg administration, suggesting REM rebound involvement. A particularly interesting finding was that while placebo changes decreased in the final quarter, the active treatment groups showed significant increases. This change suggests that daridorexant further promotes the tendency for REM sleep to increase in the latter half of sleep, and this effect persisting through the final quarter suggests that daridorexant’s sleep effects are maintained through the night.

### Promotion of natural sleep architecture

The prolonged TST observed with daridorexant administration in this study included increases not only in REM sleep but also in NREM sleep (stages N2 and N3). Basic research [24, 25] suggests that orexin neurons govern the transition from REM to NREM sleep and that DORAs may restore several abnormalities un sleep micro-architecture as well as macro-architecture. Neural circuits mediated by orexin receptors are involved in the regulation of sleep and wakefulness, and NREM and REM sleep. Daridorexant is expected to promote sleep and restore natural sleep architecture. Detailed examination of its effects on daytime mood, sleep satisfaction, cognitive function, and their relationships with sleep architecture is anticipated in the future.

### Limitations

This study has several limitations. First, this is a secondary analysis of Phase II clinical trial data, and the exploratory nature should be acknowledged. Second, the sample size was limited to 47 cases from a Japanese population, which may limit generalizability to other ethnic groups. However, consistent dose-response patterns were observed and statistically validated. Additionally, this study aimed to evaluate dose-response relationships in sleep architecture and REM sleep enhancement in the latter part of the night, rather than to determine the optimal dose through pairwise dose comparisons. The sample size in this trial was limited for detailed between-group comparisons. Future prospective studies in larger populations, including elderly patients, are needed to verify the differential effects and clinical significance of each dose. Third, direct comparisons with other hypnotics were not performed, limiting conclusions regarding relative efficacy. Fourth, subjective outcomes and next-day cognitive function assessments were not conducted, making it impossible to evaluate correlations between PSG improvements and clinical endpoints. Finally, while NAW was introduced as a novel sleep fragmentation index showing promising results, validation in larger populations would strengthen its application as a sleep assessment tool.

## Conclusion

We examined the effects of daridorexant on sleep architecture in insomnia patients using PSG data from a Phase II clinical trial conducted in Japan. The results demonstrated that daridorexant administration reduced the number of persistent awakenings, decreased sleep fragmentation, and improved sleep continuity. Daridorexant administration dose-dependently increased not only total sleep time but also NREM sleep time and REM sleep time. Particularly for NREM sleep, light sleep stage N1 decreased while dose-dependent increases were confirmed for stages N2 and N3. Furthermore, sleep quarter analysis revealed that the reduction in persistent awakenings was evident during the first quarter of sleep with 50 mg administration. REM sleep increases, considered a rebound phenomenon, were observed during the first quarter with 25 mg and 50 mg administration, with marked increases also noted in the final fourth quarter. These study results suggest that daridorexant has the potential to promote recovery toward natural sleep architecture in individuals with insomnia.

## Supplementary Information

Below is the link to the electronic supplementary material.

Supplementary Material 1

## Figures and Tables

**Table 3:**
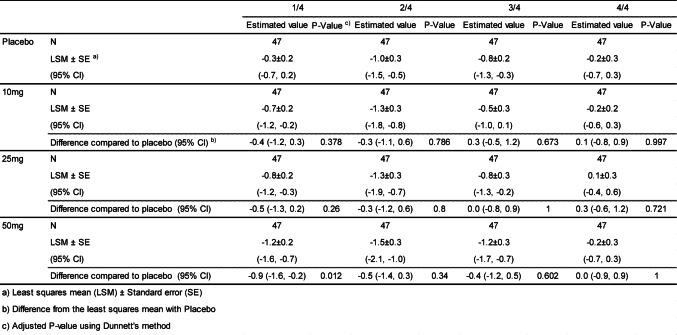
Quartile-Based changes from baseline in number of persistent awakenings: statistical comparisons between Daridorexant and placebo

**Table 4:**
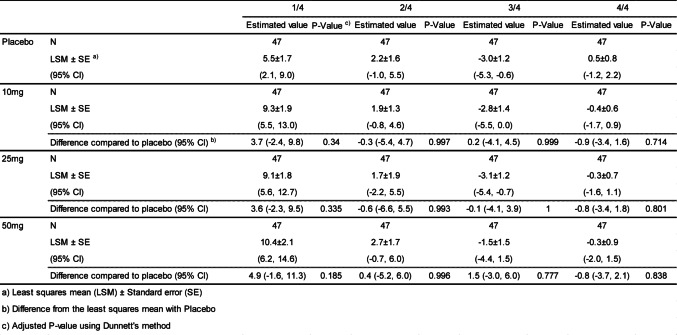
Quartile-Based changes from baseline in stage N3 sleep time: statistical comparisons between Daridorexant and placebo

**Table 5:**
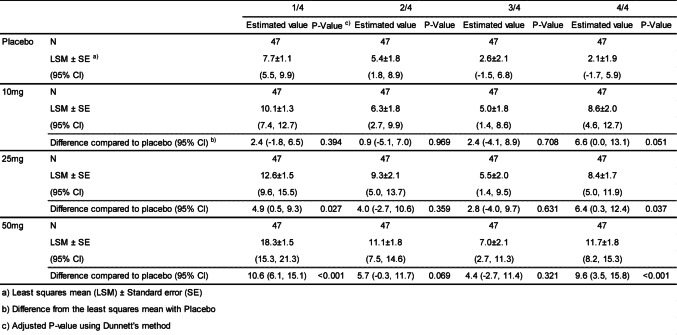
Quartile-Based changes from baseline in REM sleep time: statistical comparisons between Daridorexant and placebo

Note: These supplementary tables provide detailed statistical analyses corresponding to Figure 4, showing quartile-based temporal patterns of treatment effects throughout the 8-hour sleep recording period. Each table presents pairwise comparisons between active daridorexant doses and placebo using Dunnett’s adjustment for multiple comparisons

## Data Availability

The data analyzed in this study are proprietary and were obtained from Idorsia Pharmaceuticals Japan Ltd. under a data sharing agreement. Due to contractual restrictions, this data cannot be made publicly available.

## Appendix A. Statistical comparisons between active doses and placebo for Temporal sleep parameters

This appendix provides detailed statistical comparisons for the temporal analysis of sleep parameters presented in Figure 4. P-values were calculated using Dunnett’s method for multiple comparisons between each daridorexant dose and placebo across four quarters of the night.
