# Supplementary material for: Effects of daridorexant on sleep architecture in Japanese patients with insomnia disorder: analysis of a phase II randomized controlled trial
Source: Sleep Biol Rhythms. 2026 Feb 9;24(2):279–89. doi: 10.1007/s41105-025-00628-2 (PMC13066052; doi:10.1007/s41105-025-00628-2)
Supplement: Supplementary file 1 — Supplementary Material 1 [file 41105_2025_628_MOESM1_ESM.docx]

**Appendices**

**Appendix A. Statistical Comparisons Between Active Doses and Placebo for Temporal Sleep Parameters**

This appendix provides detailed statistical comparisons for the temporal analysis of sleep parameters presented in Figure 4. P-values were calculated using Dunnett’s method for multiple comparisons between each daridorexant dose and placebo across four quarters of the night

**Table A1. Quartile-Based Changes from Baseline in Number of Persistent Awakenings: Statistical Comparisons Between Daridorexant and Placebo**

**Table A2. Quartile-Based Changes from Baseline in Stage N3 Sleep Time: Statistical Comparisons Between Daridorexant and Placebo**


**Table A3. Quartile-Based Changes from Baseline in REM Sleep Time: Statistical Comparisons Between Daridorexant and Placebo**

***Note:****These supplementary tables provide detailed statistical analyses corresponding to Figure 4, showing quartile-based temporal patterns of treatment effects throughout the 8-hour sleep recording period. Each table presents pairwise comparisons between active daridorexant doses and placebo using Dunnett’s adjustment for multiple comparisons.*
